# Supplementary material for: Effects of Para-Toluenesulfonamide on Canine Melanoma Xenotransplants in a BALB/c Nude Mouse Model
Source: Animals (Basel). 2022 Sep 2;12(17):2272. doi: 10.3390/ani12172272 (PMC9454485; doi:10.3390/ani12172272)
Supplement: Supplementary file 1 [file animals-12-02272-s001.zip › animals-1795076-supplementary.pdf]

animals MDPI

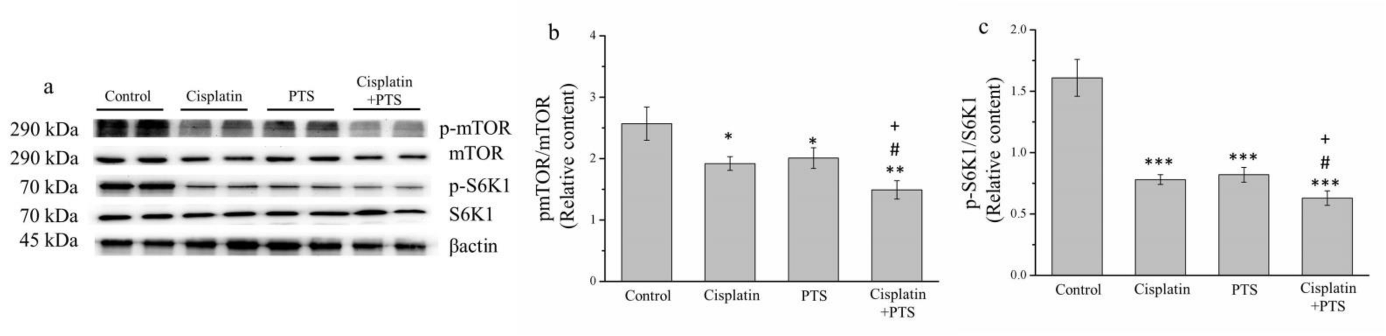

Figure S1. Western blot analysis of aspects of the mTOR signaling pathway in M5 canine melanoma tumors implanted in BALB/c nude mice. (a) Representative western blot of mTOR and S6K1 expression. (b, c) Quantification of the relative expression levels of mTOR phosphorylation (b) and S6K1 phosphorylation (c). The mice were administered the following treatments three times a week: saline (control), 2 mg/kg cisplatin (cisplatin), 100 mg/kg PTS (PTS), or 100 mg/kg PTS and 2 mg/kg cisplatin (cisplatin + PTS). Data are mean ± SD, n = 7 per group. * $P < 0.05$, ** $P < 0.01$, and ***$P < 0.001$ vs. control; # $P < 0.05$ vs. cisplatin; + $P < 0.05$ vs. PTS.

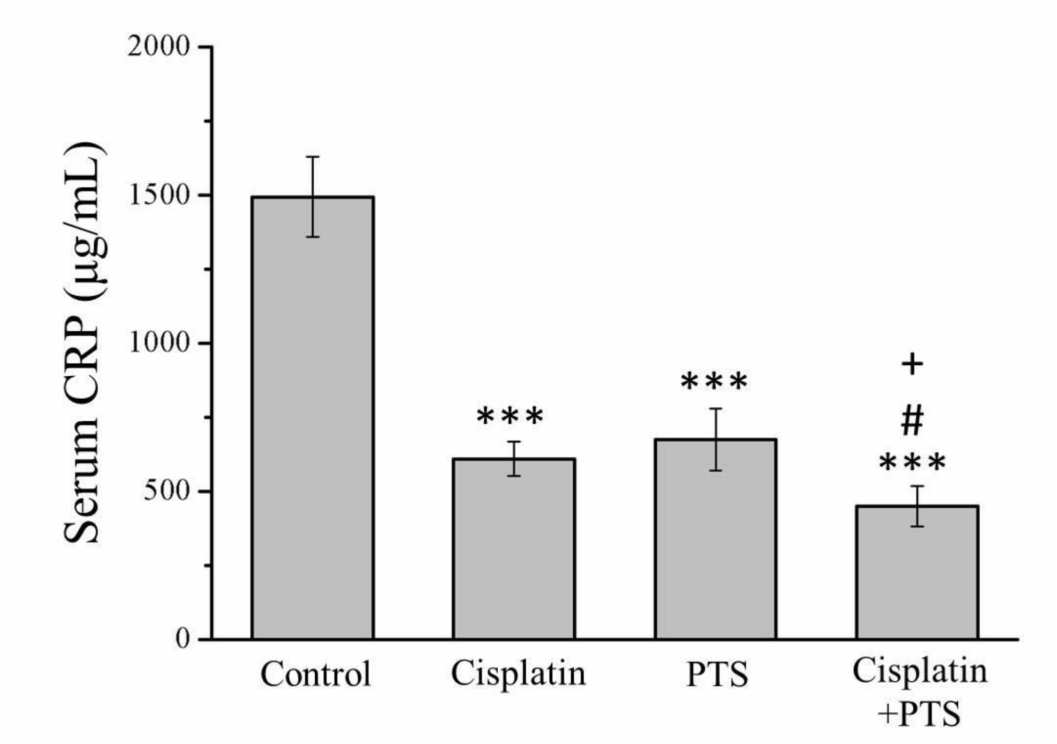

Figure S2. Serum levels of CRP in BALB/cByJNarl mice implanted with canine melanoma tumor cells. The mice were administered the following treatments three times a week: saline (control), 2 mg/kg cisplatin (cisplatin), 100 mg/kg PTS (PTS), or 100 mg/kg PTS and 2 mg/kg cisplatin (cisplatin + PTS). Data are mean ± SD, n = 7 per group. *** $P < 0.001$ vs. control; # $P < 0.05$ vs. cisplatin; + $P < 0.05$ vs. PTS.

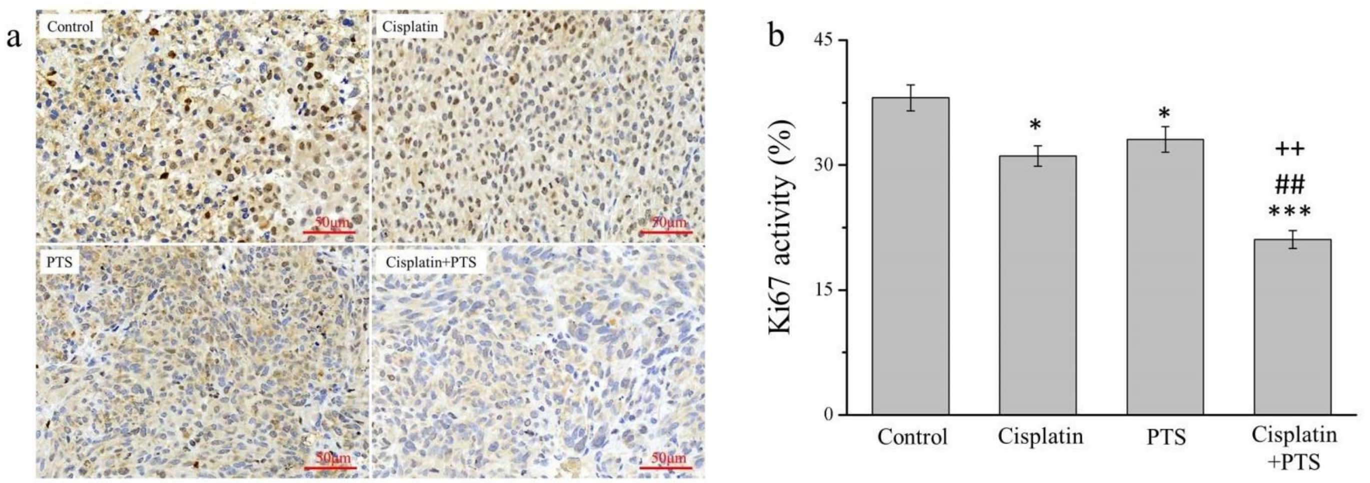

Figure S3. IHC analysis of Ki67 expression in M5 canine melanoma tumors implanted in BALB/c nude mice. (a) Representative IHC images indicating Ki67 expression. (b) Quantitative comparison of expression levels of Ki67. The mice were administered the following treatments three times a week: saline (control), 2 mg/kg cisplatin (cisplatin), 100 mg/kg PTS (PTS), or 100 mg/kg PTS and 2 mg/kg cisplatin (cisplatin + PTS). Data are mean ± SD, n = 7 per group. * $P < 0.05$ and *** $P < 0.001$ vs. control; ## $P < 0.01$ vs. cisplatin; ++ $P < 0.01$ vs. PTS. Scale bars: 50 μm.

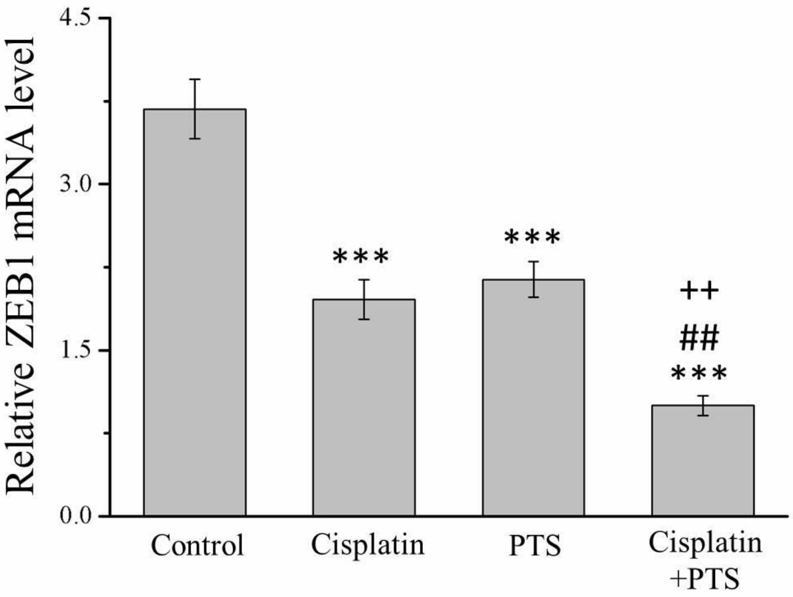

Figure S4. Quantitative PCR analysis of the relative expression levels of ZEB1 mRNA in M5 canine melanoma tumors implanted in BALB/c nude mice. The mice were administered the following treatments three times a week: saline (control), 2 mg/kg cisplatin (cisplatin), 100 mg/kg PTS (PTS), or 100 mg/kg PTS and 2 mg/kg cisplatin (cisplatin + PTS). Data are mean ± SD, n = 7 per group. *** $P < 0.001$ vs. control; ## $P < 0.01$ vs. cisplatin; ++ $P < 0.01$ vs. PTS.

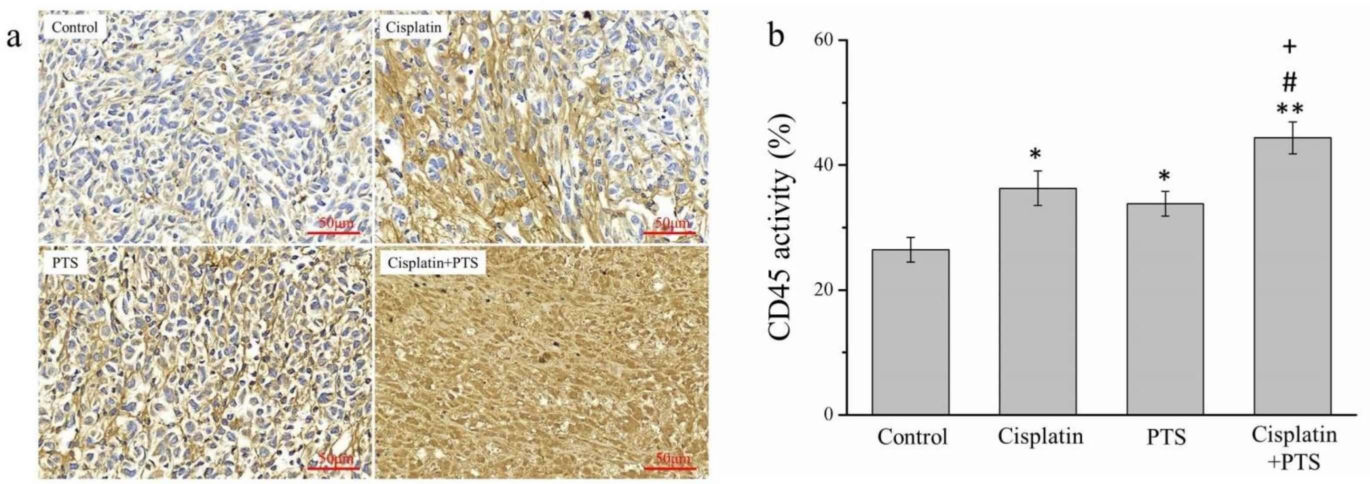

Figure S5. IHC analysis of CD45 expression in M5 canine melanoma tumors implanted in BALB/c nude mice. (a) Representative IHC images indicating Ki67 expression. (b) Quantitative comparison of expression levels of Ki67. The mice were administered the following treatments three times a week: saline (control), 2 mg/kg cisplatin (cisplatin), 100 mg/kg PTS (PTS), or 100 mg/kg PTS and 2 mg/kg cisplatin (cisplatin + PTS). Data are mean ± SD, n = 7 per group. * $P < 0.05$ and ** $P < 0.01$ vs. control; $^{\#}$ $P < 0.05$ vs. cisplatin; $^{+}$ $P < 0.05$ vs. PTS. Scale bars: 50 μm.
